# Characterizing core muscle morphometry in postpartum women with pelvic girdle pain and asymptomatic subjects: a comparative cross-sectional study

**DOI:** 10.7717/peerj.20601

**Published:** 2026-01-08

**Authors:** Ziling Lin, Bo Chen, Ruoling Chen, Xueling Chen, Yanjun Hou, Yanping Liu, Lili Lin, Zhiwei Lin, Xiangbin Wang, Cheng Zeng

**Affiliations:** 1 College of Rehabilitation Medicine, Fujian University of Traditional Chinese Medicine, Fuzhou, China; 2 Rehabilitation Technology Innovation Center by Joint Collaboration of Ministry of Education and Fujian Province, Fuzhou, China; 3 Department of Rehabilitation, Fuzhou Second General Hospital, Fuzhou, China; 4 College of Acupuncture and Massage, Fujian University of Chinese Medicine, Fuzhou, China; 5 Department of Rehabilitation, The Third People’s Hospital Affiliated to Fujian University of Chinese Medicine, Fuzhou, China; 6 Pelvic Floor Rehabilitation Specialty, Rehabilitation Hospital affiliated to Fujian University of Chinese Medicine, Fuzhou, China; 7 Department of Rehabilitation, Fujian Medical University Union Hospital, Fuzhou, China

**Keywords:** Postpartum women, Pelvic girdle pain, Musculoskeletal ultrasound, Core muscle, Active straight leg raise test, Abdominal muscles, Diaphragm muscle, Lumbar multifidus muscle

## Abstract

**Background:**

This study aimed to examine the differences in core muscle morphometry and contraction changes between postpartum individuals with and without pelvic girdle pain (PGP). Understanding the observed changes in muscle thickness and contraction characteristics is crucial for tailoring effective core muscle rehabilitation strategies that promote optimal postpartum recovery.

**Methods:**

A cross-sectional study was conducted involving 150 postpartum women with PGP and 50 asymptomatic individuals as matched controls. Real-time musculoskeletal ultrasound was used to measure core muscle morphometry and assess changes in muscle thickness and percentage change during various tasks.

**Results:**

Compared with asymptomatic women, individuals with PGP exhibited substantially reduced diaphragm excursion and thinner muscle thickness of the transverse abdominal muscle (TrA) during active straight leg raise (ASLR) tests with abdominal muscle contractions (*P* < 0.05). The TrA respiratory contraction rate and preferential activation ratio during abdominal breathing were also lower in the PGP group (PGP = 0.46 (0.13 to 1.41); asymptomatic women = 0.98 (0.05 to 2.05), *P* = 0.01). In addition, postpartum women with PGP showed increased activation of the internal oblique muscle during ASLR tests with abdominal muscle contractions compared to controls (PGP: 47%; asymptomatic women: 45%, *P* < 0.05). Furthermore, the bilateral lumbar multifidus muscle was smaller and thinner on the right side in the PGP group than the asymptomatic group (*P* < 0.001).

**Conclusion:**

Postpartum women with PGP exhibited thinner muscle thickness on core muscle morphometry and less muscle change during abdominal breathing and the ASLR test.

## Introduction

Pelvic girdle pain (PGP) refers to pain located between the posterior iliac crest and gluteal folds, specifically around the sacroiliac joints and/or pubic symphysis (Vleeming et al., 2008). PGP is a prevalent issue among pregnant and postpartum women, affecting between 20% and 65% globally (Vleeming et al., 2008). It substantially affects women’s daily lives, with research indicating that it can affect their ability to perform daily activities for up to 11 years postpartum (Elden et al., 2016; Bergstrom, Persson & Mogren, 2019).

Although hormonal and mechanical changes as well as nonoptimal muscular stabilization of the pelvic joints may partly explain PGP, its complete etiology, particularly during the postpartum period when hormonal levels have normalized, remains unknown (Vleeming et al., 2008). A literature review indicated that musculoskeletal changes are closely related to persistent postpartum PGP (Sakamoto & Gamada, 2019). Previous studies have demonstrated a significant relationship between inner core muscles and musculoskeletal pain (Majeed et al., 2019; Jeong, Kim & Park, 2023). The lumbar multifidus (MF), lateral abdominal wall, diaphragm muscle (DM), and pelvic floor muscles (PFM) collectively form a cylinder that functions synergistically to generate a network of lumbo-pelvic forces that contribute to pelvic stability (Cervera-Cano et al., 2023). However, pregnancy and childbirth can lead to changes in core muscles. The 2022 Clinical Practice Guidelines for postpartum women with PGP strongly emphasize the importance of core muscle examination as a critical component of a physiotherapist’s assessment (Simonds, Abraham & Spitznagle, 2022). Notably, muscle thickness and its contractile changes (including percentage changes) are key morphological parameters for evaluating muscle function and structural adaptations. Previous studies have established that these parameters are indicative of muscle contractility, structural integrity, and functional performance (Russo et al., 2018; Rams et al., 2024). Therefore, understanding the core muscle alterations associated with PGP during the postpartum period is vital for developing effective muscle training programs for patients with PGP.

Previous studies found patients with PGP have excessive contraction of the transverse abdominal muscles (TrA) during ASLR tests, indicating that there is no rationale for the prescription of exercises to enhance TrA contraction in PGP (Mens & Pool-Goudzwaard, 2017a), whereas some studies did not find these results (Rostami et al., 2015; Weis et al., 2017). In addition, while a study indicated that the ability to contract deep abdominal muscles and the strength of the PFM were not associated with PGP (Stuge et al., 2006), other studies found differences (Stuge, Saetre & Ingeborg Hoff, 2013; Kharaji et al., 2023). These conflicting results may be attributed to differences in patient selection, different tasks to facilitate contraction, and the extent of motor learning that occurred before the assessment. Furthermore, numerous studies have failed to distinguish between low back pain (LBP) and PGP, despite their differing symptoms and treatments. There is substantial evidence indicating that PGP is a condition distinct from LBP and should be investigated independently (Vleeming et al., 2008). They can be diagnosed and differentiated from each other by history taking, clinical examination, provocative test maneuvers, and imaging (Vleeming et al., 2008; Casagrande et al., 2015). Moreover, only one study examined the muscle thickness of MF and found no difference in resting muscle thickness between postpartum individuals with PGP and those without PGP (Chua et al., 2025). Although previous studies have focused on one or two muscles within the inner core group, none have comprehensively examined the morphological characteristics and contraction changes of all inner core muscles in postpartum individuals with PGP.

Therefore, a cross-sectional observational study should be conducted to address this research gap. This study aimed to use ultrasound-based morphometry to compare core muscle thickness and contraction changes between postpartum individuals with PGP and asymptomatic controls. Understanding these differences in muscle morphometry may have significant implications for precise core training of PGP.

## Materials & Methods

### Design

**Figure 1:**
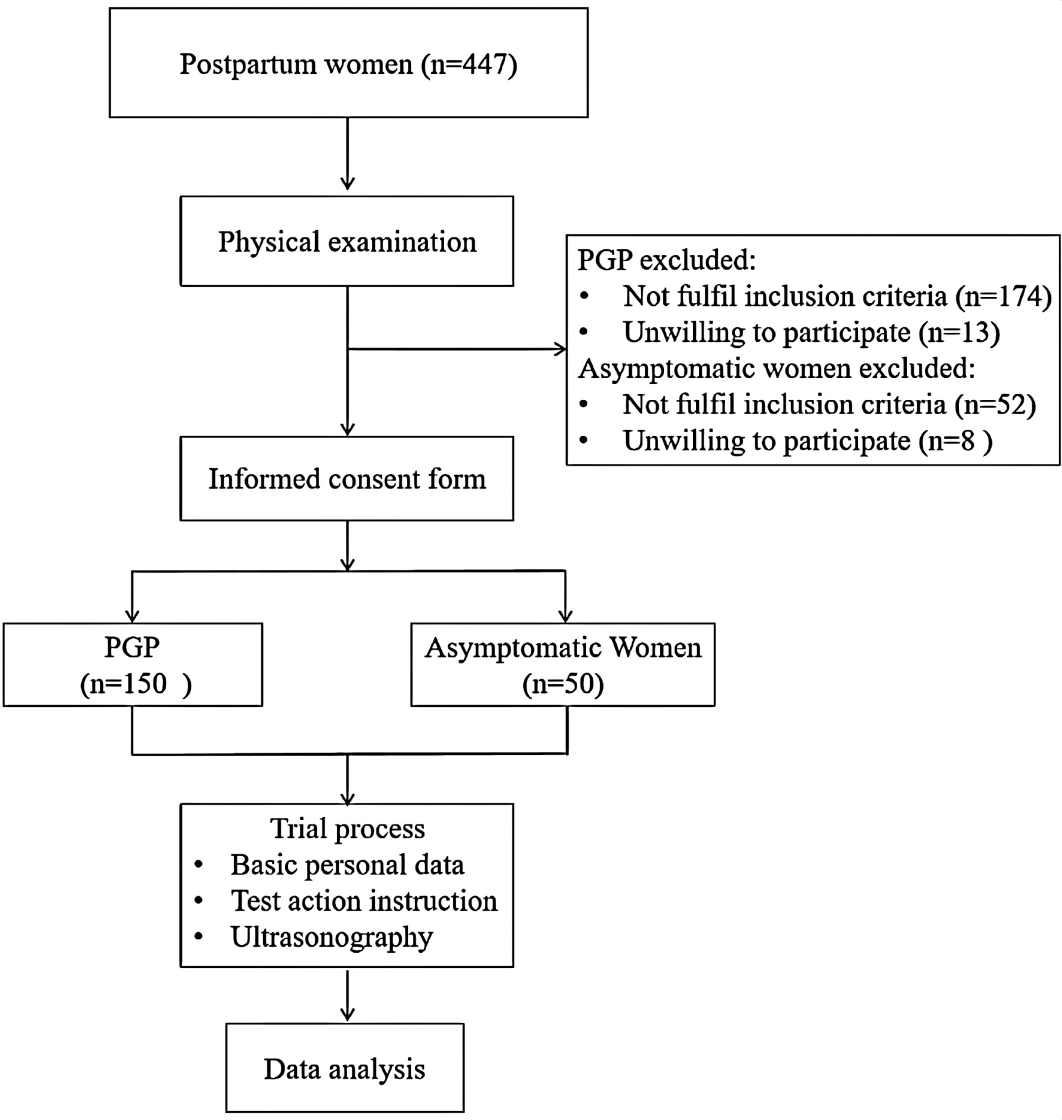
Flow chart of participants through the study.

A cross-sectional observational study was conducted on postpartum women with PGP between March 2023 and May 2024 (Fig. 1). This study was designed and reported in accordance with the Reporting of Observational Studies in Epidemiology recommendations (Von Elm et al., 2008). This study was approved by the Research Committee of Rehabilitation Hospital affiliated to Fujian University of Chinese Medicine (ethics approval number: 2023KY-001-01).

### Participants

Participants were recruited through posters and brochures displayed at rehabilitation clinics in two academic tertiary hospitals, newspaper advertisements, referrals, and neighborhood announcements. All participants were eligible if they were postpartum women aged between 20 and 40 years. An *a priori* power analysis was performed using G-power. At least 50 asymptomatic and 50 patients with PGP, were needed to ensure 80% power to detect a moderate effect size (0.60) using alpha of 0.05 in this comparative study (Bashir et al., 2019). The PGP was diagnosed based on the updated 2011 diagnostic criteria for PGP (Kanakaris, Roberts & Giannoudis, 2011), which specifies pain localized to the sacroiliac joints and/or pubic symphysis, arising during pregnancy or within the first month postpartum, and limiting activities such as rolling in bed or single-leg stance. Additionally, at least three of the following six pelvic pain provocation tests were required to be positive (Vleeming et al., 2008; Laslett et al., 2005): the posterior pelvic pain provocation (P4) test, Patrick’s Faber test, palpation of the long dorsal ligament, Gaenslen’s test, deep palpation of the symphysis and the modified Trendelenburg test of the pelvic girdle.

Eligibility for PGP group also required the following criteria (1) within six months-five years after delivery, as 78.0% of women with PGP may resolve spontaneously after birth (Bjelland et al., 2013), (2) pain attributed to pregnancy or delivery that lasts for at least 6 months; (3) Visual Analog Scale (VAS) pain score between 3 and 6 cm, the average pain score from onset to present. In contrast, the asymptomatic group had (1) no history of prior PGP and no current pain in the lumbopelvic region for at least 6 months; (2) no multiple joint pain in the extremities. The exclusion criteria for all participants included: (1) presence of pain between the subcostal and fifth lumbar regions (Casagrande et al., 2015); (2) symptoms of PGP present before pregnancy; (3) history of surgery on the lumbar spine, pelvic girdle, hip, *etc*, and (4) acute pelvic inflammation, obvious physiological defects like limb mutilation or hearing disability, major diseases like cancer, serious cardiovascular disease, and cognitive impairment that may influence this study. Informed written consent was obtained from participants after explaining the detailed procedure. All the histories, physical examinations, and basic information were recorded on a case record form, and performed face-to-face by the same physiotherapist.

### Data collection

A Siemens-Sequoia Silver ultrasound system was used for musculoskeletal ultrasound imaging. An experienced physical therapist specializing in women’s health with over 3 years of experience in musculoskeletal ultrasound conducted the assessments. Before the musculoskeletal ultrasound assessment, the participants received standardized verbal instructions for abdominal breathing and maximal contraction of the PFM. A water-soluble transmission gel was applied to the measurement site, and either the 10L4 (superficial probe) or 5C1 (convex array probe) was placed on the muscles based on the specifications outlined in Table 1 (Vellucci et al., 2018; Batibay et al., 2021; Hii et al., 2024). The intrarater reliability of the ultrasonography measurement in this study was proven to be good to excellent (the intrarater reliability = 0.875–0.974) previously in 15 healthy postpartum women, as shown in Table 2.

**Table 1:** Measurement positions of each muscle.

Muscles	Positions	Measurement sites	Parameters
Diaphragm muscle (DM)	Supine	Immediately below the right costal margin in the mid-clavicular line or in the anterior axillary line. Muscle thickness and excursion were obtained using B-mode and M-mode imaging.	R/MI/ME/DE/RCR
External oblique muscle (EO) Internal obliques muscle (IO) Transverse abdominal muscle (TrA)	Supine	A vertical position relative to the muscles from the midpoint of the line connecting the inferior angle of the last rib to the iliac crest. The hyperechoic fascia lines from top to bottom are: EO-IO-TrA.	R/MI/ME/RCR/ ASLR-NA/ASLR-A
Lumbar Multifidus muscle (MF)	Prone	The L5 vertebra lateral direction, between the facet joint and fascia.	R/MI/ME/RCR
Pelvic floor muscle (PFM)	Supine	The probe was placed in the perineum and tilted to the left and right to obtain a parasagittal section. In the parapelvic floor sagittal section, the long axis of the left and right puborectalis muscles.	R/MC/CR

**Table 2:** The mean (SD), ICC, SEM, and MDC values for core muscles thickness at rest in healthy controls for intrarater reliability.

Muscle	Mean (SD)		ICC	SEM	MDC
	First	Second			
DM	1.35 (0.53)	1.33 (0.42)	0.927	0.127	0.352
EO	2.55 (1.41)	2.66 (1.32)	0.973	0.220	0.610
IO	3.71 (1.05)	3.77 (1.02)	0.964	0.193	0.534
TrA	1.87 (0.31)	1.83 (0.37)	0.894	0.111	0.307
L-MF	16.30 (1.19)	16.06 (1.31)	0.873	0.438	1.213
R-MF	20.34 (2.91)	18.93 (2.26)	0.875	0.938	2.600
L-PFM	14.79 (2.46)	14.56 (2.74)	0.886	0.879	2.436
R-PFM	12.17 (2.70)	12.84 (3.38)	0.893	0.991	2.743

**Notes.**

DM Diaphragm muscle EO External oblique muscle IO Internal obliques muscle TrA transverse abdominal muscle L-MF Left lumbar multifidus muscle R-MF Right lumbar multifidus muscle PFM Pelvic floor muscle ICC Intraclass correlation coefficients SEM standard error of measurement MDC minimal detectable change

Muscle thickness images were acquired using B-mode ultrasound during the following activities: (1) at rest without muscle tension (R), (2) during maximum inspiration (MI) and maximum exhalation (ME), where participants fully inhaled and exhaled and then held the exhalation or apnea for at least 3 s. Muscle thickness images were acquired once at the end of each maximum inspiration and exhalation; (3) during the Active Straight Leg Raise test (ASLR), where participants lying on their back, raised their straightened left lower leg and then their right leg to a preplaced height of a 20-cm ruler, with and without abdominal muscle contractions toward the spine during the exhalation of abdominal breathing (ASLR-A/ASLR-NA); and (4) maximum contraction (MC) of pelvic floor muscle.

The diaphragm and abdominal muscles were obtained on the right side in the supine position (Zhou et al., 2023). The assessment of diaphragmatic excursion (DE) was used the 3.5 MHz curvilinear transducer, which was placed on the lower intercostal area between the midclavicular and anterior axillary lines for the right hemidiaphragm. M-mode recording was performed on consecutive abdominal breaths to accurately measure the diaphragmatic excursion at the exact points of end-inspiration and end-expiration (Ziaeifar et al., 2022).

The MF muscle was collected in the prone position, with a pillow under their abdomen to relax the lumbar muscles. Participants were instructed to follow standardized verbal commands to perform abdominal breathing, maintaining the position for 3–5 s at both end-inspiration and end-expiration. Using a transperineal measurement of the PFM, participants were instructed to empty the urinary bladder and bowel before measurement (Volloyhaug et al., 2016). And participants lay supine with their hips flexed at 60 degrees and abducted (Kim, Choi & Shin, 2015). Participants were instructed to follow standardized verbal commands to contract their PFM as if they were maximally squeezing to stop the flow of urine, holding the contraction for 3–5 s. Morphological evaluation focused on the puborectalis muscle (a key component of the PFM group) was measured for thickness at rest and during maximum contraction, and the contraction ratio subsequently calculated. The MF muscle and PFM were collected on both sides.

**Figure 2:**
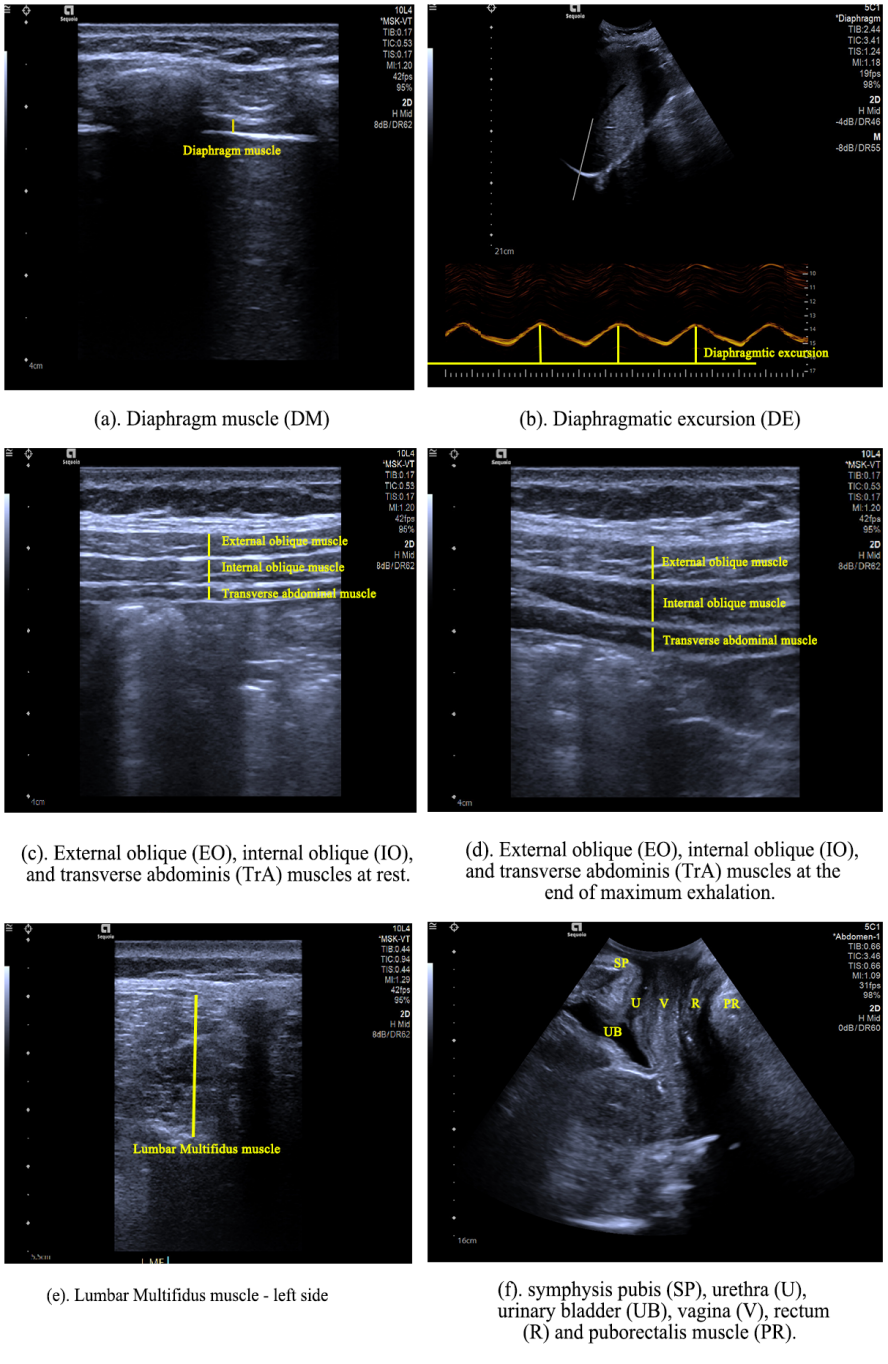
(A–F) Ultrasonographic images. (A) Diaphragm muscle (DM). (B) Diaphragmatic excursion (DE). (C) External oblique (EO), internal oblique (IO), and transverse abdominis (TrA) muscles at rest. (D) External oblique (EO), internal oblique (IO), and transverse abdominis (TrA) muscles at the end of maximum exhalation. (E) Lumbar Multifidis muscle (left side). (F) Symphysis pubis (SP), urethra (U), urinary bladder (UB), vagine (V), rectum (R) and puborectalis muscle (PR).

Participants were prevented from viewing the screen, thereby eliminating any visual sensory feedback during task performance. Each measurement was repeated three times, and the average was used to calculate the percentage change in thickness (Bashir et al., 2019). The image parameters were analyzed using MicroDicom software. Muscle thickness of the core muscles were respectively measured from the superior fascial border to the inferior fascial border at its thickest point, as shown in Figs. 2A–2F. The change and percentage of muscle thickness was calculated using the following formula (Burzynski et al., 2023):

(1) %EO (External oblique muscle) $= \frac{EO}{EO+IO+TrA} \times $ 100%;
(2) %IO (Internal obliques muscle) = $ \frac{IO}{EO+IO+TrA} \times $ 100%;
(3) %TrA (Transverse abdominal muscle) = $ \frac{TrA}{EO+IO+TrA} \times $ 100%;
(4) Respiratory contraction rate (RCR) = $ \frac{(ME-MI)}{R} $; contraction rate (CR) = $ \frac{(MC-R)}{R} $; a higher RCR or CR indicates better contraction activity.
(5) TrA preferential activation ratio (TrA PAR: difference in the TrA proportion of the total lateral abdominal muscle thickness in going from the relaxed to the contracted state) = (TrA contracted/TrA + EO + IO contracted) −(TrA at rest/TrA + EO + IO at rest) (Pulkovski et al., 2012).
(6) L/R: Comparison of muscle thickness between the left and right sides at rest. A ratio closer to 1 indicates greater symmetry, >1 indicates greater muscle thickness on the left side, and <1 indicates greater muscle thickness on the right side.

### Statistical analysis

The analysis was conducted using SPSS (Version 27). Data normality was assessed using visual inspection and the Shapiro–Wilk test. Continuous variables were reported as mean (SD) or median (first quartile (Q1) − third quartile (Q3)). Student’s *t*-test or Mann–Whitney U test for continuous data and the chi-square test for categorical data were employed to compare baseline characteristics between the two groups. Between group differences in muscle thickness values was assessed using an analysis of covariance with BMI, age, and parity as covariates as they have previously been shown to have an effect on PGP (Wuytack, Begley & Daly, 2020). *P* < 0.05 was considered statistically significant across the entire statistical analysis.

## Results

### Participant characteristics

A total of 200 participants completed the trial, with a mean age of 32.29 years (SD = 4.65). Table 3 lists the clinical characteristics. Both groups were comparable in terms of baseline characteristics. In terms of the number of deliveries, 57% of women in the PGP group and 46% in the control group had a history of single delivery, with the remaining having experienced multiple deliveries. Additionally, the majority of these deliveries were vaginal (PGP group: 67%; control group: 62%). In the PGP group, pain was predominantly located on the left side, followed by pain in both the anterior pelvic and the right side (L:49%, Both: 25%, AP:14%, R:12%). The mean duration of pain in the PGP group was 12.68 months (SD = 9.82).

**Table 3:** Between-group differences in baseline characteristics. The normally distributed variables are described as $(\bar {\mathrm{x}}\pm \mathrm{s})$, other data with non-normal distribution are expressed as median (interquartile range) M (P25, P75).

Characteristics	PGP (*n* = 150)	Asymptomatic women (*n* = 50)	Effect size	*P*
Age, mean (SD), y	32.81 (3.38)	33.40 (2.89)	0.58 (1.64 to 0.47)	0.27
Height , mean (SD), cm	1.60 (0.55)	1.60 (0.58)	0.00 (0.01 to 0.02)	0.86
Weight, Me (IQR), kg	56.55 (52.88 to 62.13)	57.70 (52.73 to 61.00)	0.00 (0.200 to 2.30)	0.91
BMI, Me (IQR), kg/m^2^	22.04 (20.32 to 24.22)	22.16 (20.70 to 23.48)	0.02 (0.84 to 0.83)	0.97
Postpartum time, Me (IQR), m	15.45 (7.76 to 32.74)	18.00 (6.88 to 39.18)	2.07 (−7.53 to 2.23)	0.36
Maximum gestational weight, Me (IQR), kg	66.00 (61.00 to 74.00)	61.75 (61.75 to 72.35)	0.00 (2.20 to 3.50)	0.76
gestational age, Me (IQR), day	273.50 (267.75 to 280.00)	275.00 (272.50 to 280.00)	1.00 (−4.00 to 1.00)	0.32
Delivery mode, n				
vaginal delivery	101	31	0.58	0.75
cesarean delivery	45	17		
Both	4	2		
Number of deliveries, n				
1	86	23	2.70	0.44
2	53	23		
3	10	3		
4	1	1		
Pain side, n				
L	74	NA	—	—
R	18			
AP	21			
Both	37			
Disease duration, mean (SD), m	12.68 (9.82)	NA	—	—
VAS, mean (SD), cm	4.5 (0.59)	NA	—	—

**Notes.**

BMI Body mass index m month L left side R right AP Anterior Pelvic Both At least two areas IQR interquartile range Me median

### Diaphragm muscle and lumbar multifidus muscle

Table 4 summarizes the between-group differences in DM and lumbar MF morphometry between women with PGP and asymptomatic women. The PGP group exhibited lower DM excursion during breathing, indicating a decreased range of motion (*P* < 0.001). There were no significant differences in DM thickness at rest, during MA, or ME between the two groups (*P* > 0.05). Furthermore, the RCR showed no significant change between the two groups (*P* = 0.36). In terms of MF muscle morphometry, the PGP group exhibited thinner muscle thicknesses at rest, during MA, and ME compared with the asymptomatic women. In addition, statistically significant differences were observed in the right RCR and left–right symmetry (*P* < 0.05). The PGP group exhibited smaller changes in muscle contractions on the right side (*P* < 0.001) and weaker MF muscles on the right side than on the left side (*P* = 0.02).

**Table 4:** Between-group differences in diaphragm and lumbar multifidus muscle morphometry. The normally distributed variables are described as $(\bar {\mathrm{x}}\pm \mathrm{s})$, other data with non-normal distribution are expressed as median (interquartile range) M (P25, P75).

Muscle	Parameters	PGP (*n* = 150)	Asymptomatic (*n* = 50)	*β*(95% CI)	*P*
DM	R (mm)	1.55 (0.47)	1.60 (0.58)	0.03 (0.19 to 0.13)	0.38
	MI (mm)	2.92 (0.62)	3.03 (0.72)	0.16 (0.40 to 0.06)	0.31
	ME (mm)	1.40 (0.40)	1.29 (0.37)	0.1 (0.02 to 0.23)	0.05
	RCR	1.11 (0.66)	1.23 (0.77)	0.10 (0.30 to 0.11)	0.36
	DE (cm)	1.65 (0.58)	1.97 (0.60)	0.3 (0.5 to 0.11)	<0.001
L-MF	L-R (mm)	16.37 (14.69 to 18.02)	20.15 (2.94)	−3.80 (−4.67 to −2.93)	<0.001
	L-MI (mm)	16.05 (14.42 to 17.93)	21.05 (2.71)	−4.70 (−5.60 to −3.86)	<0.001
	L-ME (mm)	15.83 (14.41 to 17.57)	20.33 (3.32)	−4.11 (−5.05 to −3.2)	<0.001
	L-RCR	0.06 (0.03 to 0.10)	0.07 (0.03 to 0.13)	−0.01 (−0.02 to 0.01 )	0.82
R-MF	R-R (mm)	15.04 (1.60)	19.72 (2.31)	−4.18 (−4.95 to −3.33)	<0.001
	R-MI (mm)	16.09 (14.69 to 17.87)	21.32 (2.36)	−5.07 (−5.86 to −4.34)	<0.001
	R-ME (mm)	15.79 (14.34 to 17.50)	19.83 (18.68 22.22)	−4.44 (−5.23 to −3.49)	<0.001
	R-RCR	0.06 (0.03 to 0.11)	0.09 (0.04 to 0.14)	−0.02 (−0.04 to −0.01)	<0.001
MF	L/R	1.08 (0.97 to 1.22)	1.00 (0.12)	0.67 (0.14 to 0.12)	0.02

**Notes.**

DM Diaphragm muscle L-MF Left lumbar multifidus muscle R-MF Right lumbar multifidus muscle R rest state MI maximum inspiration ME maximum exhalation RCR Respiratory contraction rate DE diaphragmatic excursion

### Abdominal muscles

Table 5 presents the differences in abdominal muscle morphometry between the groups. No statistically significant differences were observed in the EO, IO, and TrA muscles at rest, during MA, or the ASLR test without abdominal contraction (*P* > 0.05). In the PGP group, only the TrA muscle thickness during ASLR-A was significantly thinner, indicating an attenuated morphology of the deep core muscle (*P* < 0.001). In addition, the contraction rate during respiration was significantly lower than in asymptomatic women (*P* = 0.01). The TrA PAR was also significantly weaker in patients with PGP (*P* = 0.01).

**Table 5:** Between-group differences in the external oblique muscle, internal oblique muscle, and transverse abdominal muscle morphometry. The normally distributed variables are described as $(\bar {\mathrm{x}}\pm \mathrm{s})$, other data with non-normal distribution are expressed as median (interquartile range) M (P25, P75).

Muscle	Parameters	PGP (*n* = 150)	Asymptomatic (*n* = 50)	*β*(95% CI)	*P*
EO	R (mm)	3.49 (0.94)	3.63 (0.93)	−0.14 (−0.44 to 0.16)	0.41
	MI (mm)	2.81 (0.75)	2.80 (2.30 to 3.30)	−0.05(−0.30 to 0.20)	0.61
	ME (mm)	3.49 (2.87 to 4.27)	3.75 (0.92)	−0.16 (−0.50 to 0.14)	0.37
	RCR	0.22 (0.21)	0.24 (0.17)	−0.03 (−0.09 to 0.04)	0.59
	ASLR-NA (mm)	3.06 (2.59 to 3.79)	3.75 (0.92)	−0.30 (−0.60 to 0.03)	0.12
	ASLR-A (mm)	3.32 (2.73 to 3.98)	3.47 (0.94)	0.01 (−0.27 to 0.30)	0.48
IO	R (mm)	4.84 (4.14 to 5.41)	4.69 (1.30)	−0.23 (−0.17 to 0.62)	0.27
	MI (mm)	3.62 (3.17 to 4.40)	3.50 (3.01 to 4.67)	−0.04 (−0.30 to 0.35)	0.75
	ME (mm)	5.13 (4.32 to 6.00)	5.22 (4.41 to 6.27)	−0.13 (−0.59 to 0.30)	0.65
	RCR	0.27 (0.23)	0.31 (0.22)	0.04 (−0.11 to 0.38)	0.43
	ASLR-NA (mm)	4.76 (4.07 to 5.64)	5.03 (4.15 to 6.11)	−0.20 (−0.64 to 0.20)	0.40
	ASLR-A (mm)	5.29 (4.49 to 6.37)	5.6 (1.52)	−0.05 (−0.52 to 0.40)	0.93
TrA	R (mm)	1.96 (1.67 to 2.34)	2.00 (1.73 to 2.37)	−0.03 (−0.19 to 0.13)	0.98
	MI (mm)	1.57 (1.33 to 1.83)	1.67 (1.40 to 1.98)	−0.09 (−0.23 to 0.03)	0.17
	ME (mm)	2.53 (1.92 to 3.21)	2.75 (2.26 to 3.85)	−0.40 (−0.70 to 0.10)	0.15
	RCR	0.40 (0.21 to 0.79)	0.61 (0.30 to 1.09)	0.15 (0.30 to 0.02)	0.01
	ASLR-NA (mm)	2.02 (1.70 to 2.41)	2.13 (1.86 to 2.50)	−0.10 (−0.27 to 0.07)	0.26
	ASLR-A (mm)	2.93 (2.30 to 3.64)	3.48 (1.19)	−0.47 (−0.82 to 0.10)	0.01
	TrA PAR	0.46 (0.13 to 1.41)	0.98 (0.05 to 2.05)	−0.49 (−0.91 to 0.10)	0.01

**Notes.**

EO External oblique muscle IO Internal obliques muscle TrA transverse abdominal muscle R rest state MI maximum inspiration ME maximum exhalation RCR Respiratory contraction rate ASLR-NA/A Active Straight Leg Raise test without and with abdominal contraction PAR preferential activation ratio

### Abdominal muscles percentage and PFM morphometry

**Figure 3:**
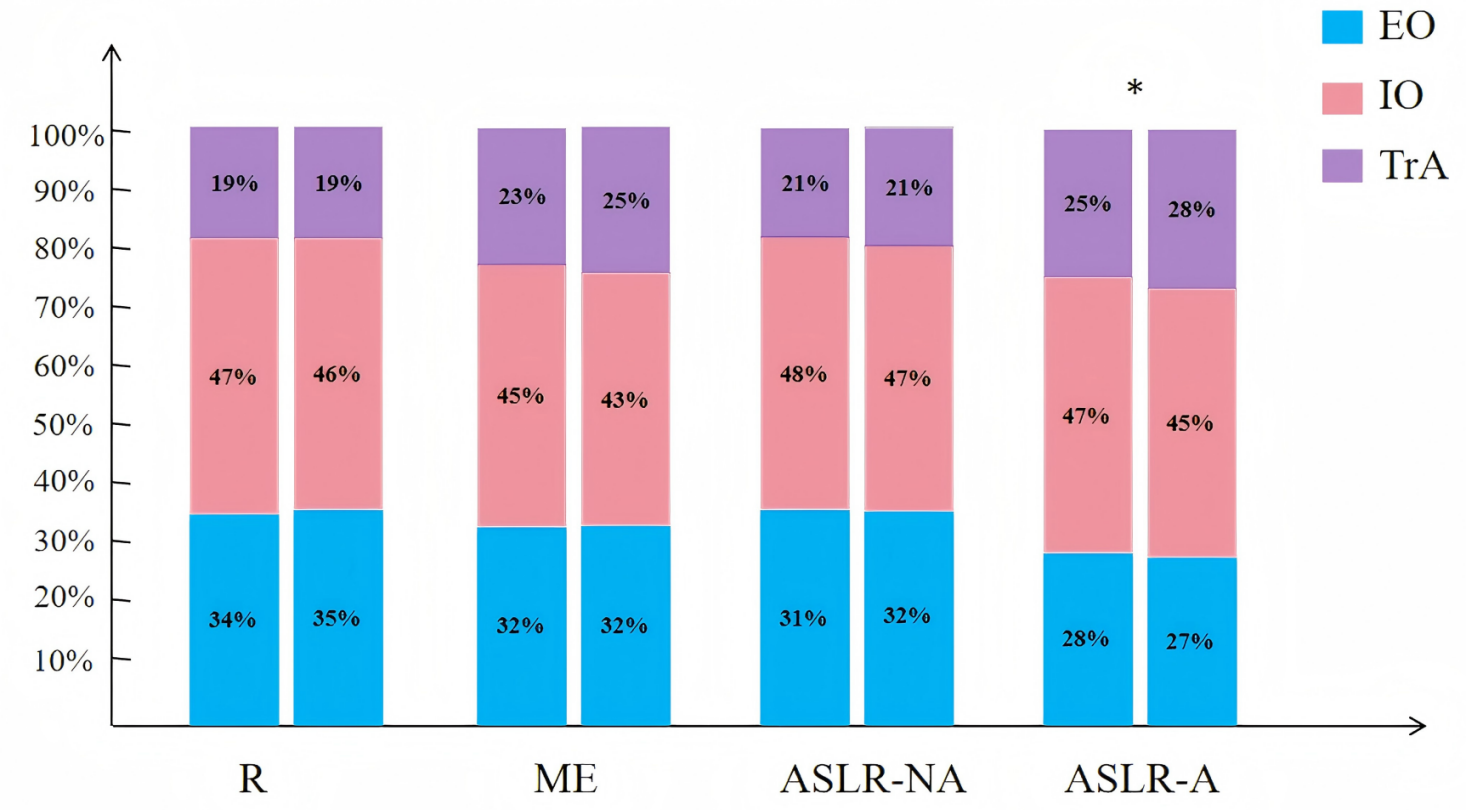
Between-group differences in the EO, IO, and TrA muscle percentage s of morphometry under the four conditions. On the left is the PGP group, and on the right is the control group; EO, External oblique muscle; IO, Internal oblique muscle; TrA, transverse abdominal muscle; ASLR- NA /A, Active Straight Leg Raise test without and with abdominal contraction. An asterisk (*) indicated that differences existed between groups.

Figure 3 shows the percentage differences in the three abdominal muscles (EO, IO, and TrA) at rest, during ME, and the ASLR test with or without abdominal contractions. No statistical differences were observed between the muscles at rest, ME, and ASLR-NA conditions. However, the PGP group exhibited greater activation of the IO and reduced TrA contraction during ASLR-A condition (*P* < 0.05). Table 6 presents that no significant differences were observed in the PFM morphometry between the two groups (*P* >0.05).

## Discussion

This study found that participants with PGP exhibited differences in core muscle morphometry. Compared with asymptomatic women, those with PGP exhibited reduced DE and thinner TrA during ME and the ASLR-A state. In contrast, the IO muscle showed greater activation. Furthermore, the preferential activation of the TrA muscle during respiration was weaker, with more thin and asymmetrical lumbar multifidus muscles in the PGP group.

### Diaphragm muscle

Our findings revealed that DE was significantly lower in the PGP group, although there were no significant differences in muscle thickness or changes in thickness between the two groups. This reduction in DE is consistent with previous reports showing decreased diaphragmatic excursion during respiratory tasks in postpartum women with lumbopelvic pain (Kharaji et al., 2023). The diaphragm is a crucial part of the “core” deep trunk muscle group. It plays a vital role in contributing to spinal stiffness through the influence of intra-abdominal pressure, mechanical effects, and the attachments of the diaphragm crura. Consequently, there is increasing recognition in the literature of the crucial relationship between respiratory function and musculoskeletal pain (Beeckmans et al., 2016; Mohan, Paungmali & Sitilertpisan, 2018). The findings of this study indicate that diaphragmatic mobility may be significantly restricted in postpartum individuals with PGP. Training to improve the mobility of diaphragm may help improve core function in postpartum women with PGP.

### Abdominal muscles

This study found that TrA thickness during ME, respiratory muscle contraction ratio, and ASLR test with abdominal contraction were lower in women with PGP. In addition, TrA PAR was reduced compared with the control group. Most previous studies reported no difference in the resting thickness of these muscles between individuals with LBP or pregnancy-related PGP (Stuge et al., 2006). Furthermore, no significant differences in abdominal muscle thickness were observed between women who experienced back pain during pregnancy and those who did not (Weis et al., 2017). These results are consistent with existing literature regarding the EO, IO, and TrA muscles at rest. Nevertheless, different findings regarding dynamic muscle contraction changes have emerged. Mens & Pool-Goudzwaard (2017b) reported that in patients with PGP, TrA exhibited minimal contraction during pain-free tests, whereas excessive contraction was observed during pain-provoking tests. Excessive TrA contraction was also observed during the ASLR test in patients with long-lasting pregnancy-related posterior PGP (Mens & Pool-Goudzwaard, 2017a). Conversely, in participants with a history of LBP or chronic low back pain, the percentage of change in TrA thickness was lower than that in the control group (Seyed Hoseinpoor et al., 2015; Sutherlin et al., 2018). In this study, participants with PGP exhibited weaker TrA contraction during the ASLR test with abdominal contraction.

**Table 6:** Between-group differences in pelvic floor muscle morphometry. The normally distributed variables are described as $(\bar {\mathrm{x}}\pm \mathrm{s})$, other data with non-normal distribution are expressed as median (interquartile range) M (P25, P75).

Muscle	Parameters	PGP (*n* = 150)	Asymptomatic (*n* = 50)	*β*(95% CI)	*P*
L-PFM	R (mm)	12.98 (11.28 to 15.52)	14.64 (10.73 to 14.64)	1.04 (2.39 to 0.22)	0.40
	MC (mm)	14.72 (3.22)	15.70 (3.99)	0.98 (−2.08 to 0.12 )	0.09
	CR	10.56 (8.56 to 13.10)	12.05 (3.46)	1.00 (−2.13 to 0.00)	0.30
R-PFM	R (mm)	11.02 (9.25 to 13.48)	11.88 (9.53 to 16.23)	0.82 (−2.05 to 0.31)	0.15
	MC (mm)	11.15 (9.98 to 11.50)	10.70 (9.85 to 11.80)	0.04 (−0.02 to 0.10)	0.98
	CR	1.06 (0.96 to 1.18 )	1.03 (0.95 to 1.20 )	0.02 (−0.04 to 0.08)	0.54

**Notes.**

PFM Pelvic floor muscle R rest state MC maximum contraction CR contraction rate

Furthermore, although there was no difference in the IO% at rest, the IO% during the ASLR test with abdominal contraction was significantly higher in the PGP group than in the asymptomatic group. Previous research has reported increased activity of the oblique abdominal muscles in individuals with lumbopelvic pain (Beales, O’Sullivan & Briffa, 2009; Hodges et al., 2009; Whittaker, Warner & Stokes, 2013). Recent studies have also indicated a significant association between IO thickness and lumbopelvic pain, indicating that changes in IO thickness may be associated with lumbopelvic pain during the second trimester of pregnancy (Casagrande et al., 2015; Kawabe et al., 2023). The findings of this study indicate that women with PGP may experience pelvic hypermobility or pain, leading to increased activity of the IO muscle, increasing intra-abdominal pressure, and maintaining spinal pelvic stability. In contrast, the TrA, a transverse muscle that may be inhibited by an enlarged fetus during pregnancy, can become weaker and thinner in individuals with PGP (Fukano et al., 2021). Therefore, further research is required to explore the roles of the TrA and IO in the abdominal wall and their impact on the development and persistence of PGP. Clinically, physical therapists should identify the different abdominal muscle contractions during breathing exercises and core stabilization training, especially focusing on contractions of the TrA.

### Lumbar multifidus muscle

Regarding the MF muscles, this study found that women with PGP had thinner MF muscle thicknesses on both sides compared with asymptomatic women. In addition, the right side exhibited lower contraction changes during respiration and was thinner than the left side. Previous systematic reviews and meta-analyses have reported that patients with LBP often have smaller MF muscles with considerable intramuscular fat infiltration (Seyedhoseinpoor et al., 2022). However, these studies did not focus on patients with PGP. A prospective study using magnetic resonance imaging observed that individuals with pregnancy-related lumbopelvic pain had a smaller cross-sectional area of paraspinal muscles on the affected side and greater muscle asymmetry than the healthy group (Long et al., 2021). Nevertheless, a recent study reported no significant difference in MF thickness at rest among women with PGP (Chua et al., 2025). This discrepancy may be due to differences in delivery mode; Chua et al. (2025) only recruited participants with vaginal births, whereas this study included participants with vaginal and cesarean births, potentially affecting the results.

Combined with previous research findings, the reduced thickness of the MF muscle may be attributed to disuse atrophy resulting from pain stimulation (Wan et al., 2015; Xu et al., 2016). In this study, the muscles on the right side were thinner and exhibited less contraction in individuals with PGP, likely due to two factors: first, in the anatomically posterior oblique chain, the gluteus maximus is connected by the fascia to the sacroiliac joint with the latissimus dorsi on the opposite side. Pain predominantly on the left side of PGP (49%, as presented in Table 3) can lead to muscle spasms and inhibit the gluteus maximus on the painful side. Compensatory hypertrophy may develop on the nonpainful side, where the latissimus dorsi is hypertrophic and suppresses deeper MF muscles. Second, radiographic studies have indicated that individuals with sacroiliac joint pain often exhibit increased anterior pelvic rotation during ASLR compared with healthy individuals (Mens et al., 1999), with more pronounced anterior rotation on the right side (Gibbons, 2017). The right vertical spinal muscles are more susceptible to compensatory tension as the right pelvic rotation increases. Consequently, patients with PGP exhibit increased average left-side thickness in this study. These asymmetrical muscular compositions can result in abnormal biomechanics during segmental movements (Long et al., 2021). Thus, attention to the MF muscle and its symmetry is crucial in rehabilitation assessment and treatment. In the future, the relaxation of the superficial muscle group and the symmetry of core muscle training can be combined through treatment.

### Pelvic floor muscle

The PFM morphological parameters did not significantly differ between the two groups in this study. This is consistent with previous research by Stuge, Saetre & Ingeborg Hoff (2013), which reported no significant differences in PFM thickness between PGP and controls during rest and automatic contractions during ASLR. Similarly, other studies have reported no differences in PFM strength and endurance in women with or without PGP, although poor ability in PFM pre-activation was found in those with PGP (Sjodahl et al., 2016; Starzec-Proserpio et al., 2022). Another study identified a correlation between PGP and deep PFM tenderness during pregnancy, although no significant difference in muscle strength was observed compared with controls (Fitzgerald & Mallinson, 2012). These results indicate that although there may be no difference in muscle thickness, there was a change in the tenderness point of muscle tension between the PGP and the controls. The lack of significant differences could also be attributed to the varied delivery modes among the postpartum women in this study. Further research into muscle tenderness and pre-activation during tasks is also necessary.

### Limitations

This study had several limitations. First, although different delivery modes were classified, they were not included in the analysis, which may have influenced the PFM outcomes. Second, all participants who voluntarily enrolled in this study reported moderate pain and mild disability, which may limit the generalizability of the findings to individuals with more severe symptoms. Future studies should include a broader spectrum of symptoms. Finally, this study did not combine the function of external core muscles, such as the erector spinalis, gluteus maximus, and gluteus medius. Further studies are required to examine the performance of key internal and external core muscles under various loading tasks. Based on these morphological differences in the core muscle, investigating the long-term effects of targeted muscle training is also worth further research in the future.

## Conclusions

In conclusion, this study enhances our understanding of core muscle morphological changes in postpartum women with PGP. The results of this study indicate that women with PGP exhibit reduced diaphragmatic excursion, altered abdominal muscle function, and thinner lumbar multifidus muscles compared to asymptomatic controls. These findings support and build upon existing research, revealing that PGP has unique core muscle morphology and contraction characteristics. This insight could contribute to the development of more effective postnatal exercise programs. Treatment modalities that focus on diaphragmatic motion and emphasize TrA and MF contractions during breathing or exercise training may have important implications for women who have developed PGP during pregnancy or after delivery.

## Supplemental Information

10.7717/peerj.20601/supp-1 Supplemental Information 1 Clinical Research Protocol

10.7717/peerj.20601/supp-2 Supplemental Information 2 STROBE checklist

10.7717/peerj.20601/supp-3 Supplemental Information 3 Raw data Basic data: Group: 1 (trial group); 2 (control group)
Groups: 1 (trial group); 2 (control group)
Delivery mode: : 1:vaginal delivery; 2: cesarean delivery ; 3: both 1 and 2
DM data: Group: 1 (trial group); 2 (control group)
AM data: Group: 1 (trial group); 2 (control group)
MT data: Group: 1 (trial group); 2 (control group)
PFM data: Group: 1 (trial group); 2 (control group)

## Additional Information and Declarations

### Data Availability

The following information was supplied regarding data availability: The raw data is available in the Supplemental File.

### Clinical Trial Registration

The following information was supplied regarding Clinical Trial registration: CTR2400079630.
