# Supplementary material for: Characterizing core muscle morphometry in postpartum women with pelvic girdle pain and asymptomatic subjects: a comparative cross-sectional study
Source: PeerJ. 2026 Jan 8;14:e20601. doi: 10.7717/peerj.20601 (PMC12790787; doi:10.7717/peerj.20601)
Supplement: Supplemental Information 1 [file peerj-14-20601-s001.pdf]

Clinical Research Protocol

1. Research Background

Pelvic girdle pain (PGP) refers to pain in the inguinal region, posterior iliac crest, and gluteal fold, particularly near the sacroiliac joints. It may radiate to the posterior thigh or present alone at the pubic symphysis. Epidemiological data show that 20% of pregnant women experience pelvic girdle dysfunction, with 93% reporting persistent symptoms within three months postpartum and 21% still experiencing pain two years after delivery. PGP is more prevalent than low back pain in pregnant women and is associated with higher rates of functional impairment. Daily activities such as walking, standing, or sitting can trigger or exacerbate PGP, with symptoms intensifying after just 30 minutes of activity, thereby limiting daily life and social participation. Persistent pain during pregnancy or postpartum may induce fear, anxiety, and even chronic pain syndrome. Accurate assessment of PGP and enhanced rehabilitation interventions are therefore critical for maternal health under China's three-child policy.

PGP arises from biomechanical alterations during pregnancy, including weight gain, postural changes, increased intra-abdominal pressure, and structural shifts in the spine and pelvis. Pelvic asymmetry may affect ligament tension (mechanical closure ability), while core muscle dysfunction and motor control disorders disrupt force closure, both major contributors to PGP onset or exacerbation. Preliminary clinical observations indicate that PGP patients exhibit pelvic asymmetry and core muscle dysfunction during static and dynamic states. However, these findings rely on manual assessments by experienced pelvic floor rehabilitation experts, lacking standardized, objective data to characterize their musculoskeletal dysfunction. Thus, precise evaluation of core muscle function and pelvic symmetry during static/dynamic states is essential.

Musculoskeletal ultrasound, a safe, cost-effective, and accessible technique, offers unique sensitivity and accuracy in assessing musculoskeletal systems. Unlike other muscle evaluation methods, it measures comprehensive structural parameters, including muscle length, thickness, cross-sectional area, fascicle angle, and pennation angle. Real-time ultrasound monitoring of muscle contraction demonstrates higher reliability than electromyography (EMG) or MRI, with intraclass correlation coefficients (ICCs) of 0.89–0.96 for repeated measurements.

This study aims to collect musculoskeletal ultrasound data of core muscles in postpartum PGP patients to characterize morphological changes, inform the development and optimization of rehabilitation protocols, and provide objective references for outcome and prognostic evaluation.

2. Subject Recruitment

Eligible subjects will be recruited from the Affiliated Rehabilitation Hospital and the Affiliated Third People's Hospital of Fujian University of Traditional Chinese Medicine. Recruitment will include two groups: an asymptomatic control group and a PGP patient group.

Inclusion Criteria for PGP group

- within six months-five years after delivery
- pain attributed to pregnancy or delivery that lasts for at least 6 months
- Visual Analog Scale (VAS) pain score between 3 and 6 cm, the average pain score from onset to present

Inclusion Criteria for asymptomatic group

- no history of prior PGP and no current pain in the lumbopelvic region for at least 6 months
- no multiple joint pain in the extremities

Exclusion Criteria

- Pregnant or lactating women
- presence of pain between the subcostal and fifth lumbar regions
- symptoms of PGP present before pregnancy
- history of surgery on the lumbar spine, pelvic girdle, hip, etc,
- acute pelvic inflammation, obvious physiological defects like limb mutilation or hearing disability, major diseases like cancer, serious cardiovascular disease, and cognitive impairment that may influence this study.

Withdrawal/Exclusion Criteria

Subjects who meet inclusion criteria, sign the informed consent form, but discontinue participation prematurely (voluntarily or involuntarily) due to the following reasons will be considered withdrawn or excluded:

- Misenrollment due to non-compliance with inclusion criteria;
- Incomplete clinical data affecting safety assessment;
- Refusal to undergo required evaluations;
- Voluntary withdrawal by the subject;
- Severe adverse events (e.g., falls, allergies);
- Other circumstances deemed appropriate for termination by researchers.

Researchers must document the reasons for termination and record outcome indicators at the time of withdrawal, especially for subjects who voluntarily withdraw.

3. Outcome Measures and Assessment Methods

Before the musculoskeletal ultrasound assessment, the participants received instructions on performing abdominal respiration, proper muscle contraction, and relaxation techniques. A water-soluble transmission gel was applied to the measurement site, and either the 10L4 (superficial probe) or 5C1 (convex array probe) was placed on the muscles based on the specifications outlined.

Diaphragmatic muscle: Immediately below the right costal margin in the mid-clavicular line or in the anterior axillary line. Muscle thickness and excursion were obtained using B-mode and M-mode imaging.

External oblique muscle, Internal obliques muscle, Transverse abdominal muscle: A vertical position relative to the muscles from the midpoint of the line connecting the inferior angle of the last rib to the iliac crest. The hyperechoic fascia lines from top to bottom are: EO-IO-TrA.

Lumbar Multifidus muscle: The L5 vertebra lateral direction, between the facet joint and fascia.

Pelvic floor muscle: The probe was placed in the perineum and tilted to the left and right to obtain a parasagittal section. In the parapelvic floor sagittal section, the long axis of the left and right puborectalis muscles.

Muscle thickness was measured during the following activities: (1) at rest without muscle tension (R) (2) during maximum aspiration (MA) and maximum exhalation (ME), where participants fully inhaled and exhaled and then held the exhalation or apnea for at least 3 s. Muscle thickness images were acquired once at the end of each maximum aspiration and exhalation; (3) during the Active Straight Leg Raise test (ASLR), where participants lying on their back, raised their straightened left lower leg and then their right leg to a preplaced height of a 20-cm ruler, with and without abdominal muscle contractions toward the spine during the exhalation of abdominal breathing (ASLR-A/ASLR-NA); (4) during diaphragm excursion (DE) assessed using M mode under natural breathing; and (5) maximum contraction (MC) of pelvic floor muscle. The diaphragm and abdominal muscles were obtained on the right side in the supine position. The MF muscle was collected in the prone position with a pillow under their abdomen. Using a transvaginal measurement of the PFM, participants were instructed to empty the bladder and intestines before measurement. And participants lay supine with their hips and knees flexed at 60 degrees. The MF muscle and PFM were collected on both sides. Participants were prevented from viewing the screen, thereby eliminating any visual sensory feedback during task performance. Each measurement was repeated three times, and the average was used to calculate the percentage change in thickness.

4. Withdrawal and Termination Criteria

4.1 Subject Withdrawal

4.1.1 Researcher-Decided Withdrawal

Subjects will be withdrawn if:

1. Severe adverse events occur, precluding continued participation;
2. Subjects withdraw voluntarily;
3. Other circumstances render the subject unsuitable for the study.

4.1.2 Subject-Initiated Withdrawal

Subjects have the right to withdraw at any time per the informed consent form. "Withdrawal" includes unplanned loss to follow-up. Researchers should document withdrawal reasons (e.g., inability to continue due to scheduling).

4.1.3 Data Retention for Withdrawn Subjects

All records of withdrawn subjects, including outcome indicators, will be retained.

4.2 Study Termination

The entire study may be terminated prematurely if:

1. Severe unexpected adverse events (e.g., severe allergies, symptom exacerbation) occur;
2. Subjects develop serious complications or rapidly deteriorating conditions;
3. Critical flaws in the study design or implementation compromise data validity.

Termination must be promptly reported to all relevant parties (subjects, ethics committee, institutions).

5. Data Management and Traceability

5.1 Data Management

5.1.1 Case Report Forms (CRFs)

CRFs for all enrolled subjects are the property of the sponsor and may not be disclosed to third parties without authorization.

5.1.2 Database Construction

- Double data entry will be performed using independent software, with automatic error-checking and corrections.
- Queries on ambiguous data will be forwarded to researchers via clinical monitors for verification.
- A 10% random sample of CRFs will undergo manual review for accuracy.
- The database will be locked by the principal investigator, data manager, and statistician after confirming correctness, with no subsequent modifications allowed.

5.1.3 Data Storage

All research materials (subject eligibility records, CRFs, informed consent forms) will be retained by the researcher for at least 5 years after study completion.

5.2 Statistical Analysis

Data will be analyzed using SPSS 25.0. Normally distributed continuous data will be reported as mean ± standard deviation ($\bar{x} \pm s$), non-normally distributed data as median, and categorical data as frequency (proportion). Between-group comparisons will use independent t-tests or nonparametric tests. Statistical significance is set at $\alpha = 0.05$ ($P < 0.05$).

6. Quality Control and Assurance

6.1 Informed Consent Quality Control

- Post-consent, 20% of subjects will be randomly interviewed to verify understanding of the study.

- If partial or no understanding is identified, researchers will provide additional education, followed by a second 40%.

6.2 Enrollment Quality Control

Subjects will be strictly screened per inclusion/exclusion criteria:

- Eligible subjects (meeting inclusion, excluding exclusion criteria) proceed;
- Ineligible subjects are excluded with recorded reasons.

6.3 Assessment Method Quality Control

- All assessors will undergo standardized training to ensure consistent data collection and scoring.
- CRFs must be completely and accurately filled, with minimal alterations; repeated measurements by the same assessor are preferred.
- Data entry will be cross-checked by two reviewers against original records.

6.4 Statistical Analysis Quality Control

- Missing values, especially for safety indicators, are minimized. Non-recordable results (zero or undetected) must be clearly marked, not left blank.
- Missing safety-related data will prompt immediate subject recall for review； cases with critical missing values will be excluded.

6.5 Data Management Quality Control

A checklist of mandatory files will be maintained by the project manager, with missing items promptly addressed.

7. Adverse Event Management

Any adverse events during rehabilitation assessment (e.g., ultrasound gel allergy, falls) must be documented in adverse event forms, including symptoms, onset time, severity, duration, interventions, and outcomes. Reports will be submitted to the medical affairs department, scientific research department, and ethics committee. Immediate actions include:

- Ceasing the procedure, removing equipment (e.g., gel), and referring the subject to specialized care.

8. Research Ethics

This protocol must receive approval from the Ethics Committee of Fujian University of Traditional Chinese Medicine Affiliated Rehabilitation Hospital before implementation. The committee may approve, approve with modifications, reject, or suspend the study.

All subjects will receive detailed information about the study (purpose, nature, potential benefits, risks) and provide voluntary written informed consent prior to enrollment, in compliance with the Declaration of Helsinki and Good Clinical Practice (GCP) guidelines.

This translation adheres to the formal, structured style required for medical journal submissions, ensuring clarity, technical accuracy, and compliance with ethical and methodological standards.
