# Supplementary material for: Characterizing core muscle morphometry in postpartum women with pelvic girdle pain and asymptomatic subjects: a comparative cross-sectional study
Source: PeerJ. 2026 Jan 8;14:e20601. doi: 10.7717/peerj.20601 (PMC12790787; doi:10.7717/peerj.20601)
Supplement: Supplemental Information 2 [file peerj-14-20601-s002.pdf]

STROBE Statement—checklist of items that should be included in reports of observational studies

	Item No	Recommendation
**Title and abstract**	1	(*a*) Indicate the study's design with a commonly used term in the title or the abstract (Line 1-4).
		(*b*) Provide in the abstract an informative and balanced summary of what was done and what was found (Line 27-46).
**Introduction**		
Background/rationale	2	Explain the scientific background and rationale for the investigation being reported. (Line 51-96)
Objectives	3	State specific objectives, including any prespecified hypotheses. (Line 92-96)
**Methods**		
Study design	4	Present key elements of study design early in the paper (Line 100-102).
Setting	5	Describe the setting, locations, and relevant dates, including periods of recruitment, exposure, follow-up, and data collection (Line 100-125)
Participants	6	(*a*) *Cross-sectional study*—Give the eligibility criteria, and the sources and methods of selection of participants. (Line 108-122)
		(*b*) *Cohort study*—For matched studies, give matching criteria and number of exposed and unexposed. (N/A)<br>*Case-control study*—For matched studies, give matching criteria and the number of controls per case. (N/A)
Variables	7	Clearly define all outcomes, exposures, predictors, potential confounders, and effect modifiers. Give diagnostic criteria, if applicable. (Line 109-122).
Data sources/ measurement	8*	For each variable of interest, give sources of data and details of methods of assessment (measurement). Describe comparability of assessment methods if there is more than one group. (Line 126-170).
Bias	9	Describe any efforts to address potential sources of bias. (Line 176-178).
Study size	10	Explain how the study size was arrived at (N/A)
Quantitative variables	11	Explain how quantitative variables were handled in the analyses. If applicable, describe which groupings were chosen and why (Line 172 - 179)
Statistical methods	12	(*a*) Describe all statistical methods, including those used to control for confounding. (Line 172-179)
		(*b*) Describe any methods used to examine subgroups and interactions. (Line 172-179)
		(*c*) Explain how missing data were addressed. (N/A)
		(*d*) *Cross-sectional study*—If applicable, describe analytical methods taking account of sampling strategy. (N/A)
		(*e*) Describe any sensitivity analyses. (N/A)
**Results**		
Participants	13*	(a) Report numbers of individuals at each stage of study—eg numbers potentially eligible, examined for eligibility, confirmed eligible, included in the study, completing follow-up, and analysed. (Line 183-189)
		(b) Give reasons for non-participation at each stage. (Figure 1)
		(c) Consider use of a flow diagram. (Figure 1)
Descriptive data	14*	(a) Give characteristics of study participants (eg demographic, clinical, social) and information on exposures and potential confounders. (Table 1)
		(b) Indicate number of participants with missing data for each variable of interest. (Table 2)

		(c) *Cohort study*—Summarise follow-up time (eg, average and total amount). (N/A)
Outcome data	15*	*Cohort study*—Report numbers of outcome events or summary measures over time. (N/A)
		Case-control study—Report numbers in each exposure category, or summary measures of exposure. (N/A)
		Cross-sectional study—Report numbers of outcome events or summary measures. (Table 2)
Main results	16	(*a*) Give unadjusted estimates and, if applicable, confounder-adjusted estimates and their precision (eg, 95% confidence interval). Make clear which confounders were adjusted for and why they were included. (Table 3-5)
		(*b*) Report category boundaries when continuous variables were categorized. (Table 3-5)
		(*c*) If relevant, consider translating estimates of relative risk into absolute risk for a meaningful time period. (N/A)
Other analyses	17	Report other analyses done—eg analyses of subgroups and interactions, and sensitivity analyses. (N/A)
**Discussion**		
Key results	18	Summarise key results with reference to study objectives. (Line 218-222)
Limitations	19	Discuss limitations of the study, taking into account sources of potential bias or imprecision. Discuss both direction and magnitude of any potential bias. (Line 316-325)
Interpretation	20	Give a cautious overall interpretation of results considering objectives, limitations, multiplicity of analyses, results from similar studies, and other relevant evidence. (Line 223-314)
Generalisability	21	Discuss the generalisability (external validity) of the study results. (Line 327-336)
**Other information**		
Funding	22	Give the source of funding and the role of the funders for the present study and, if applicable, for the original study on which the present article is based. (Line 344-347)
